# The molecular mechanism and challenge of targeting XPO1 in treatment of relapsed and refractory myeloma

**DOI:** 10.1016/j.tranon.2022.101448

**Published:** 2022-06-01

**Authors:** Mark Sellin, Stephanie Berg, Patrick Hagen, Jiwang Zhang

**Affiliations:** a Department of Cancer Biology, Oncology Institute, Cardinal Bernardin Cancer Center, Loyola University Medical Center, Loyola University Chicago, USA; b Loyola University Chicago, Department of Cancer Biology and Internal Medicine, Cardinal Bernardin Cancer Center, Stritch School of Medicine, Maywood, IL, USA; c Department of Medicine, Division of Hematology/Oncology, Cardinal Bernardin Cancer Center, Loyola University Medical Center, Maywood, IL USA; d Department of Cancer Biology, Oncology Institute, Cardinal Bernardin Cancer Center, Loyola University Medical Center, USA

**Keywords:** Relapsed/refractory, Multiple myeloma, XPO1, Selinexor, ASCT, autologous stem cell transplantation, CDC, complement-dependent cytotoxicity, ADCC, antibody-dependent cell-mediated cytotoxicity, ADCP, antibody-dependent cellular phagocytosis, BCMA, B-cell maturation antigen:, BM, bone marrow, CBC20/80, cap-binding proteins 20 and 80, CNAs, copy-number alterations, CRM1, chromosome maintenance 1 protein, dara, daratumumab, DLBCL, diffuse large B-cell lymphoma, DOR, duration of response, FDA, food and drug administration, HD, hyperdiploid, IMiD, immunomodulators, mAb, monoclonal antibody, MM, multiple myeloma, MGUS, monoclonal gammopathy of undetermined significance, NCCN, national comprehensive cancer network, NDMM, newly-diagnosed multiple myeloma, NES, nuclear export signal, NHD, non-hyperdiploid, NXF1, nuclear RNA export factor 1, ORR, overall response rate, OS, overall survival, PFS, progression-free survival, PHAX, phosphorylated adaptor of RNA export, PIs, proteasome inhibitors, R-ISS, revised international staging system, Ran, ras-related nuclear protein GTPase, RCC1, regulator of chromosome condensation 1, RRMM, relapsed and refractory multiple myeloma, SINE, selective inhibitors of nuclear export, SPHK1, sphingosine kinase 1, TKI, tyrosine kinase inhibitor, TOP2A, topoisomerase 2?, UPR, unfolded protein response, XPO1, exportin 1

## Abstract

Multiple myeloma (MM) treatment regimens have vastly improved since the introduction of immunomodulators, proteasome inhibitors, and anti-CD38 monoclonal antibodies; however, MM is considered an incurable disease due to inevitable relapse and acquired drug resistance. Understanding the molecular mechanism by which drug resistance is acquired will help create novel strategies to prevent relapse and help develop novel therapeutics to treat relapsed/refractory (RR)-MM patients. Currently, only homozygous deletion/mutation of TP53 gene due to “double-hits” on Chromosome 17p region is consistently associated with a poor prognosis. The exciting discovery of XPO1 overexpression and mislocalization of its cargos in the RR-MM cells has led to a novel treatment options. Clinical studies have demonstrated that the XPO1 inhibitor selinexor can restore sensitivity of RR-MM to PIs and dexamethasone. We will elaborate on the problems of MM treatment strategies and discuss the mechanism and challenges of using XPO1 inhibitors in RR-MM therapies while deliberating potential solutions.

## Highlights

• Significant progress has been made on the treatment of MM during past two decades.
• Acquired drug-resistance continues to drive early relapse in primary refractory MM.
• XPO1 over-expression and cargo mislocalization are associated with drug-resistance.
• XPO1 inhibitor selinexor restores drug sensitivity to subsets of RR-MM cells.

## Introduction

Multiple myeloma is an acquired malignant plasma cell disorder that typically develops late in life, having a median age at diagnosis of 69 years. Although it is a rare disorder accounting for just 1.8% of all new cancer cases in the United States (US) and a lifetime risk of just 0.76%, it is the second most common hematological malignancy. Furthermore, due to an aging US population, a lack of curative therapy, and improved outcomes overall, the prevalence of MM is increasing [1]. In the last two decades, there has been a rapid development of novel classes of drugs, including proteasome inhibitors (PIs), immunomodulators (IMiDs), monoclonal antibodies, and immunotherapies such as bi-specifics and chimeric antigen receptor T-cell therapy (CAR-T). With the advent of new therapeutics and the increasing utilization of high-dose melphalan and autologous stem cell transplantation (ASCT) [2], 5- and 10-year overall survivals (OS) have improved across all age, race, and ethnic groups [3]. In the year 2000, the estimated 5-year OS for newly-diagnosed MM (ND-MM) patients was 35.6%, while in 2018 it was 56.6% [1]. With novel combination therapy, the progression-free survival (PFS) of relapsed refractory (RR)-MM has improved and is now frequently greater than two years [9]. While MM patients have seen significant improvements in short- and long-term outcomes, these benefits are more tempered in those with high-risk disease with R-ISS stage III patients having only a 24% 5-year PFS and 40% 5-year OS [4]. Overall, MM is still an incurable disease with only 10–15% of MM patients achieving or exceeding expected survival compared to the matched general population.

During the last decade, significant efforts have been made to understand both the genomics of the disease as well as the molecular mechanisms of drug resistance in MM. One of the major observations is the aberrant subcellular localization of proteins in RR-MM cells, specifically the increased cytosolic accumulation of nuclear exportin cargoes. Such aberrant localization of proteins is associated with increased expression and activity of the nuclear export protein exportin 1 (XPO1), making XPO1 an attractive therapeutic target for RR-MM treatment. In this paper, we will review the recent advances in our understanding of resistance mechanisms to the most commonly utilized therapeutics. We will focus specifically on XPO1 inhibition as a novel target that has led to the approval of KPT 330 (or selinexor) in the treatment of RR-MM. XPO1 inhibition in general will be explored as an avenue to overcome resistance, particularly for high-risk RR-MM patients who continue to have dismal outcomes.

## Current advances and challenges in the treatment of multiple myeloma

### Treatments for ND-MM patients

During past two decades, significant improvements in the treatment of ND-MM have been achieved. The advent of modern induction began with the introduction of IMiDs, namely thalidomide, in the late 1990s. Subsequently, the therapeutic landscape evolved rapidly with the development of multiple agents within drug classes which include IMiDs (thalidomide, lenalidomide, pomalidomide, and iberdomide), PIs (bortezomib, carfilzomib, and ixazomib), and monoclonal antibodies (daratumumab, isatuximab, and elotuzumab).

**Fig. 1:**
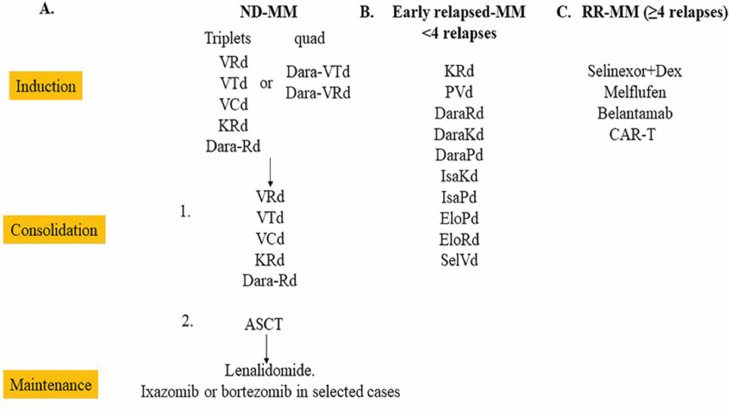
Current treatments for ND-MM (A), early relapsed MM (B) and RR-MM (C). VRd (Bortezomib, Lenalidomide, and Dexamethasone); VTd (Bortezomib, thalidomide, dexamethasone); VCd (Bortezomib, cyclophosphamide, dexamethasone); Dara-VTd (VTd plus daratumumab); Dara-VRd (VRd plus daratumumab); KRd (carfilzomib, lenalidomide and dexamethasone); autologous stem cell transplantation (ASCT); PVd (pomalidomide, bortezomib, dexamethasone); DaraRd (daratumumab, lenalidomide and dexamethasone); DaraKd (daratumumab, carfilzomib, dexamethasone); DaraPd (daratumumab, pomalidomide, dexamethasone); IsaKd (Isatuximab, carfilzomib, dexamethasone); IsaPd (Isatuximab, pomalidomide, dexamethasone); EloPd (elotuzumab, pomalidomide, dexamethasone); EloRd (elotuzumab, lenalidomide and dexamethasone) and SelVd (selinexor, bortezomib, dexamethasone).

Current standard induction regimens for ND-MM patients (regardless of bone marrow transplant eligibility) includes several triplet combinations: VRd (bortezomib, lenalidomide, and dexamethasone), VTd (bortezomib, thalidomide, and dexamethasone), VCd (bortezomib, cyclophosphamide, and dexamethasone) and KRd (carfilzomib, lenalidomide, and dexamethasone) (Fig. 1A). Among them, the most commonly used treatment combination is VRd which was established as the standard of care and lead to an improved PFS (44 v 29 months; *p =* 0.003) and OS (median not-reached v 69 months; *p =* 0.0114) compared to Rd (lenalidomide and dexamethasone) [5]. Other triplet combinations, including the recently completed MAI study evaluating DaraRD (daratumumab, lenalidomide, and dexamethasone) have produced promising results [[6], [7], [8], [9]]; however, randomized clinical trials demonstrated that no single triplet combination has been shown to be superior in terms of either PFS or OS. With the advent of better-tolerated and extremely active daratumumab-based therapy, quadruplicate-based inductions are increasingly evaluated in ND-MM patients. The recently completed GRIFFIN study comparing VRD to VRD plus daratumumab (Dara-VRD) in induction, as well as in consolidation following up front ASCT, lead to an impressive minimal residual disease (MRD – 10^−5^) rate of 51% in the Dara-RVD compared to just 20.4% in the RVD arm (*p <* 0.0001) with median PFS and OS not yet reached.

ASCT is the standard consolidative approach following induction therapy in eligible patients with ND-MM. Several international randomized trials have compared up-front ASCT and non-ASCT therapy, including the EMN02/H095,[10] EMN-411 [11], RV-MM-2019 [12], and IFM/DFCI 2009 [13]. All of these studies have shown consistent improvement in median PFS in the 1-2-year range, with some studies showing improved OS [11,12]. After modern induction therapy, ASCT consolidation can enforce a deep remission in many cases [14]. The median PFS in up-front ASCT-eligible patients now easily exceeds 4 years. However, for those patients who are not eligible for transplantation, additional cycles of triplet or quad combinations are recommended for consolidation therapy.

Post-consolidation, lenalidomide maintenance is standard therapy until the development of progressive disease based on several randomized trials, including but not limited to the IFM-2009 trial [13] and the CALGB​(Alliance) 100104 trial [15]. Maintenance therapy consistently shows improved PFS in the 18 months to 2-year range and frequently shows improvement in OS as demonstrated by a recent meta-analyses [16] With the ability to detect low-level disease using MRD analyses, there is optimism that some MM patients may be effectively cured. Thus, there is hope to develop a response-based de-escalation of therapy for these MRD-negative cases. This approach is currently being evaluated in both the MASTER trial (NCT03224507) [17] where preliminary results look promising, as well as the ongoing SWOG1803 DRAMMATIC study evaluating both doublet vs single agent maintenance therapy and maintenance discontinuation dependent on MRD negativity 2 years post-ASCT [18].

### Treatments for RR-MM patients

Despite advances in the treatment of ND-MM, relapse is inevitable for most patients even for those who achieve deep remission. Relapse is primarily due to the acquired resistance of the MM cells to the drugs used in maintenance regimens; therefore, the triplet combinations used in initial induction therapy may still be effective for most first-relapse cases. However, for second- and third-relapsed cases, myeloma tumor cells might also acquire resistance to the drugs used in induction and consolidation treatments; thus, it is recommended that the new triplet combinations for relapsed patients include 2 drugs to which the patient is not refractory towards (Fig. 1B and C). Many triplet regimens containing daratumumab or carfilzomib have been extensively studied [[19], [20], [21]]. For example, in the APSIRE trial [9], KRd in 1st relapse showed an impressive median PFS of 26.3 months, whereas DaraRd (Rd and daratumumab) triplet regimen in the POLLUX trial [22] and DaraKd (daratumumab, carfilzomib, and dexamethasone) triplet regimen in CANDOR trial showed an impressive PFS of 44.3 months and not reached, respectively [23]. Furthermore, quad-based regimens are increasingly being explored in the relapsed setting as well. Nevertheless, increasing efficacy requires careful balance with toxicity and cost-effectiveness. For these heavily pre-treated RR-MM patients, novel treatment agents with different molecular mechanisms of action to those administered prior are urgently needed.

Recently, several new drug classes have been approved by the FDA as later lines of therapies for RR-MM, including the following: (1) a lipophilic peptide-conjugated alkylator melphalan flufenamide (melflufen); (2) anti-BCMA antibody drug conjugates belantamab; (3) a novel BCMA targeted chimeric antigen receptor T-cell product (CAR-T) idecel; (4) anti-SLAMF7 monoclonal antibody elotuzumab; (5) a non-selective histone deacetylase inhibitor panobinostat; and (6) an XPO1 inhibitor selinexor [[24], [25], [26], [27], [28], [29], [30], [31], [32]]. Several pomalidomide-containing triplet combinations, including PVd (pomalidomide, bortezomib, and dexamethasone), IsaPd (isatuximab, pomalidomide, and dexamethasone), EloPd (elotuzumab, pomalidomide, and dexamethasone), and DaraPd (daratumumab, pomalidomide, and dexamethasone), have been evaluated in multiple clinical trials [[33], [34], [35]] (Fig. 1B). All of these combinations have shown very promising clinical effects, the results of which have been discussed by other review papers [36,37].

Overexpression of XPO1 has been linked to an increase in MM bone disease, relapse, and poor clinical outcomes. Selinexor represents a novel treatment for RR-MM with a complete novel mechanism distinct from all conventional treatments [[38], [39], [40], [41]]. In the following sections, we discuss the potential molecular mechanisms by which tumor cells become resistant to conventional therapeutics; we will also focus on the mechanisms by which selinexor restores drug sensitivity in RR-MM cells.

## The mechanisms underlying resistance of MM cell to conventional treatment medications

**Fig. 2:**
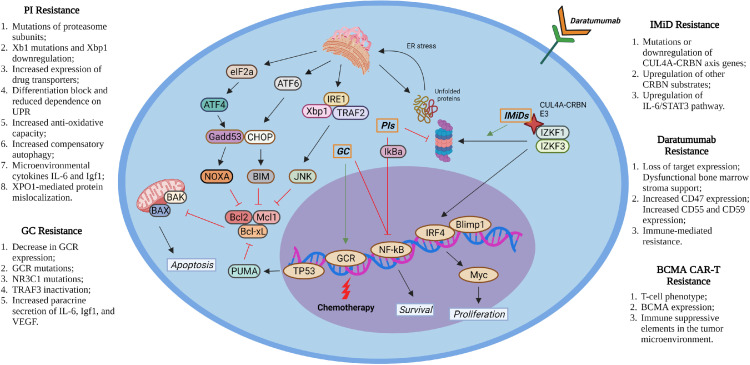
Molecular mechanisms by which the conventional drugs kill MM cells and potential mechanisms explain drug-resistance. PIs function through inhibition of the proteasome to induce ER-stress/UPR and repress NF-κB signaling. IMiDs function by inducing CRBN-mediated IKZF1/3 degradation. GC kills MM cells by inducing the expression of pro-apoptotic genes and repressing the expression of pro-survival genes through activation of GCR and inactivation of NF-κB. Chemotherapy drugs kill MM cells by inducing DNA damage, while antibody drugs kill MM cells by recognizing specific surface antigens and inducing complement-mediated cell lysis. The potential mechanisms of drug-resistance are listed. Figure created with BioRender.com.

Given the diverse pathways targeted and mechanisms of action of the various myeloma drug classes, the resistance mechanisms leading to relapse varies depending on the therapyemployed. This in turn is why 3- and 4-drug combinations synergistically kill MM cells but can still overcome drug resistance [42,43] (Fig. 2).

### The molecular mechanisms underlying PI resistance

Different from most other cell types, the malignant plasma cells in MM patients produce a large amount of immunoglobulins which make such cells highly dependent on proteasome-mediated degradation of misfolded proteins for their survival. PIs selectively inhibit the proteasomal degradation of misfolded proteins in malignant plasma cells by directly targeting the catalytic subunits (PSMB5 for bortezomib and ixazomib; PSMB2 for carfilzomib) [44,45]. The accumulation of misfolded proteins induces an unfolded protein response (UPR). ATF6a, IRE1-XBP1s, and PERK-eIF2a, as well as three UPR pathways that have been found to mediate the UPR. In normal conditions, activation of these 3 pathways induce an adaptive UPR to prevent cellular death by optimizing protein folding and ER quality control. Such adaptive responses are mediated by inducing IRE1-dependent decay of mRNA (RIDD), repressing eIF2a-mediated global translation, stimulating ER-associated protein degradation (ERAD), and/or activating ATF4-mediated selective gene expression. However, sustained activation of these pathways will result in asevere, prolonged ER-stress and will subsequently lead to cellular apoptosis by stimulating a maladaptive UPR-related JNK-BID, as well as CHOP apoptotic signaling [[46], [47], [48], [49]]. In addition, PIs also inhibits NF-κB-mediated survival signaling by preventing the degradation of the negative-regulators of NF-κB, such as IκBα.

Studies suggest that acquired PI resistance of MM cells is primarily due to the mutations or loss of proteasome subunit gene expression, and/or IRE1-XBP1 pathway gene expression. For example, mutations of proteasome subunit genes, including PSMB5, PSMB8 and PSMD1, have been reported in PI refractory MM patients [45,50,51]. Downregulation of 19S proteasome subunits and/or overexpression of PSMB5 and PSMA3 are also suggested to induce PI resistance [[52], [53], [54], [55], [56], [57], [58]]. Additionally, XBP1 mutations and XBP1 downregulation have been identified in refractory MM patients and are associated with PIs resistance [59,60]. Furthermore, increased expression of drug transporters such as ABCB1 and microenvironmental proteins (such as interleukin-6 and insulin growth factor-1) are also implicated in resistance [61,62]. Other mechanisms are associated with PI resistance, including a cellular shift of MM cells to a less-differentiated state and, as they are less-dependent on UPR, a metabolic shift with increased anti-oxidative capacity alongside a heightened compensatory autophagy.

Activating KRAS, NRAS, and BRAF mutants enhance proteasome capacity and reduce ER stress in MM through a MEK/MAPK/ELK (ETS domain-containing protein)-1/POMP pathway [63]. Interestingly, it was found that mutations in NRAS (but not KRAS) were associated with bortezomib resistance [64,65]. The molecular mechanism of this resistance still needs to be determined.

### The molecular mechanisms underlying IMiD resistance

IZKF1 (Ikaros) and IZKF3 (Aiolos) are two essential transcription factors for plasma cell differentiation. In MM cells, IKZF1 and IKZF3 upregulate IRF4 and c-MYC, which form a positive autoregulatory loop necessary for survival and proliferation [66]. The levels of IKZF1 and IKZF3 are controlled by Cereblon (CRBN)-DDB1-CRL4-axis-mediated ubiquitination and proteasomal degradation machinery. IMiDs kill MM cells by interacting with CRBN at residue 391, which disrupts protein homeostasis by inducing the degradation of IZKF1 and IZKF3 [[67], [68], [69], [70], [71], [72], [73], [74]]. IMiDs also carry numerous effects on T-cells/NK cells and contribute to anti-tumor effects by modulating the immune microenvironment in an anti-angiogenic-inflammatory manner [75,76,99,100]. Mutation or downregulation of CRBN-Ikaros axis genes (including CRBN, CUL4B, IRF4, and IKZF1), as well as upregulation of the IL-6/STAT3 pathway, have been associated with IMiD resistance [70,74,[77], [78], [79], [80], [81], [82], [83], [84]]. PTPRD is a phosphatase of STAT3 that represses its activity. PTPRD mutations promote STAT3 signaling from interleukin-6 and IMiD resistance [85]. In addition, upregulation of other CRBN substrates such as RUNX1/3 are also implicated in IMiD resistance. These substrates prevent IKZF1/3 degradation by competing with CRBN for the binding of IKZF1/3 [86].

### The molecular mechanisms underlying resistance of monoclonal antibodies

Antibody drugs kill malignant plasma cells by recognizing the specific cell surface proteins to induce complement-mediated cell lysis. Although several targets have been evaluated in the treatment of MM including CD20 [87], Interleuken-6 (siltuximab) [88], CS1/SLAMF7 [89], BCMA (SGNBCMA-001) [90], and many others [91], CD38 is by far the most developed with the FDA approval of both daratumumab (utilized in the newly-diagnosed and relapsed setting) and isatuximab (relapsed setting only). Recently, more studies have focused on targeting BCMA for RR-MM treatment due to its selective expression in both normal and tumor plasma cells [92,93]. Belantamab, an anti-BCMA antibody-drug conjugate, received FDA approval in August 2020 for the treatment of RR-MM patients who have previously received at least 4 therapies including PIs, IMiDs, and anti-CD38 monoclonal antibodies. Similar to most other antibody-drugs, anti-CD38 and anti-BCMA antibody-drugs exert anti-MM activity via antibody-dependent cell-mediated cytotoxicity (ADCC), antibody-dependent cellular phagocytosis (ADCP), complement-dependent cytotoxicity (CDC), and immunomodulatory effects. Deregulation of these pleiotropic mechanisms may cause resistance development. For example, loss of CD38 expression (either low at baseline or downregulation with therapy) has been shown to lead to daratumumab resistance and disease relapse [94]. There is also data demonstrating that daratumumab resistance can be mediated via reduction of ADCC efficacy through dysfunctional bone marrow stroma cell support that can be overcome by small-molecular inhibition of survivin by YM155 [95]. ADCP-mediated resistance may occur via up-expression of CD47 via the binding of CD47 to the signal-regulatory protein alpha (SIRPα) on tumor-associated macrophages [96]. The CD47/SIRPα complex acts as a “don't eat me” signal that induces SIRPα phosphorylation and association to Src-homology phosphatase 1 domain (SHP-1) on macrophages, resulting in the inhibition of phagocytosis [97]. This “do not eat me” signal is the focus for different mAb therapies that can block CD47 [98]. CDC resistance may be mediated by an increase in the expression of complement inhibitory proteins such as CD55 and CD59 [94]. Finally, there is increasing data showing that immune-mediated resistance leads to relapse with RNA-sequencing of BMSCs cells depleted of CD138^+^ MM cells which exhibit a different gene expression profile between progressed and daratumumab-naïve patients [97].

### The molecular mechanisms underlying resistance of chimeric antigen receptor T-Cell therapy

A relatively-recent therapeutic advancement, BCMA directed CAR-T cell therapy, has shown impressive results in MM. Despite rapid- and deep-remissions in heavily pre-treated patients, relapse is inevitable, and the mechanisms of resistance/relapse are being evaluated [99]. CAR-T cell intrinsic factors have been implicated, including the percentage of CD8^+^ T-cells with a naïve or stem memory phenotype which correlates with better outcomes [100]. MM cell intrinsic factors have also been shown to impact relapse risk, including BCMA down-expression that has demonstrated a correlation with relapse following BMCA [[100], [101], [102]]. Finally, immunosuppressive elements commonly present in the MM cell tumor microenvironment may play a role in relapse;this is being exploited by combining CAR T-Cell therapy with conventional myeloma treatments to augment T-cell function [103]. The beneficial effect of IMiDs on T-cells have been pre-clinically confirmed on CAR-T cells, as well as with providing a strong biological rationale to explore the combination of CAR-T cell + IMiDs [104].

### The molecular mechanisms underlying corticosteroid resistance

The glucocorticoids (GC), such as dexamethasone, kill MM cells by inducing pro-apoptotic signaling and repressing anti-apoptotic and metabolic pathways. GC play such a role by binding GC receptors (GCRs) which then translocate to the nucleus to modulate gene expression [105,106]. Resistance to GC is associated with a decrease in GR expression and GCR NR3C1 mutations [107,108]. Additionally, an increased tumor environment paracrine secretion of IL-6, IGF, and VEGF has also been associated with GC resistance.

### The molecular mechanisms underlying alkylating agent resistance

Chemotherapies such as melphalan kill MM cells through alkylation to induce DNA damage. Increased drug efflux, increased expression of DNA repair factors, and TP53 mutations are all associated with chemotherapy-refractory disease and relapse [109,110].

Two common strategies exist to address the issue of drug resistance in MM. First, in response to drug resistance resulting from the mutations of drug substrates, next-generation drugs were developed in order to target different interaction sites and surfaces of the mutant substrates or new substrate targets. For example, bortezomib resistance can be due to either by mutation or by over-expression of PSMB5 and next generation PIs such as Ixazomib and carfilzomib were developed. Ixazomib targets PSMB5 on an interaction site distinct from bortezomib, while carfilzomib targets PSMB2 [44,45]. Disease progression following anti-CD38 monoclonal antibody treatment, novel drugs such as anti-BCMA antibody were developed. Secondly, drug resistance resulting from activation of compensation pathways combination therapies are used to inhibit the compensation machinery. For example, activation of the IL-6/STAT3 pathway is commonly associated with IMiD resistance. Thus, a combination of an IMiD with a STAT3 inhibitor is in development [85]. If disease progression occurs after CAR-T cell therapy, possible resistance mechanisms may be due to the upregulation of immune checkpoints; thus, the addition of immune checkpoint inhibitors have been evaluated in clinical trials. Nevertheless, almost all of these therapies kill tumor cells by inducing mitochondrial apoptosis, which is at least partially TP53-dependent. This explains why many patients at time of relapse or refractory disease develop a “double-hit” of their TP53 gene (biallelic TP53 mutations/deletions), and incorporating novel therapies such as XPO1 inhibition that can kill tumor cells by inducing TP53-independent types of death are necessary [111].

## Targeting XPO1 for RR-MM treatment

Molecules greater than 40 kDa, including larger proteins, RNAs, and other biological moieties, rely on energy-dependent specialized carriers: karyopherin family proteins (including inportins and exportins) which can transport through the nuclear pore complex. The activities of such carriers are regulated by the RCC1-Ran axis [111,112]. Among 7 well-defined exportins, XPO1 (also known as chromosome maintenance 1 protein, CRM1) is the only one that functions through recognizing specific nuclear export signals (NES) on the cargoes [113]. In addition, XPO1 is selectively-responsive to a subset of RNA exports. Unbiased genetic screens using CRISPR and RNAi library screens have validated XPO1 as a therapeutic target in sarcoma, diffuse large B-cell lymphoma (DLBCL), MM, and KRAS-mutant lung cancer [[114], [115], [116]]. Selinexor (KPT-330) is one of the SINE (selective inhibitors of nuclear export) compounds and is FDA approved in both relapsed MM and DLBCL [40].

### Molecular cargoes of XPO1

At least 221 NES-containing nuclear proteins have been identified and can be found in the NESdb at http://prodata.swmed.edu/LRNes. XPO1 is responsible for transporting all these NES-containing proteins from the nucleus to the cytoplasm. Many of these proteins are tumor suppressors and cell cycle negative-regulators, including p53, RB1, p21, p27, BRCA1, BRCA2, CEBPα, PAR4, NPM1, PU.1, PP2A, MDM2, FoxOs, GCR, TOP2A, Fbw7, and DDR. Other XPO1 cargoes include proteins involved in signaling mediators (such as APC in Wnt/β-catenin and IκBα in NF-κB), growth regulators (KIT, EGFR, FLT3, BRAF, RAS-PI3K/AKT, PTEN, Ras-GRF1, and BCR-ABL), cell survival (survivin, cIAP1, MCL1, Igfbp2, Nrf2, and TERT), cell cycle positive regulators (CDK1 and cyclin B1/D1), autophagy (beclin 1, centrin, STK38, and YAP1), and others including PPAR-γ and SNAIL [117,118]. mRNA is primarily exported by the NXF1-mediated pathway, while miRNA and tRNA precursors are primarily exported by XPO5 and XPO3, respectively [119,120]. XPO1 preferentially exports ribosomal RNA (60S and 40S subunits), regulating ribosomal biogenesis. In addition, XPO1 is responsible for selectively transporting certain subsets of mRNAs, miRNA, and snRNA through exporting several RNA-binding proteins and adaptor proteins [119,120]. For example, XPO1: (1) selectively mediates the alternative export of both m^7^G-capped mRNAs and snRNA by binding to CBC20/80 [121]; (2) preferentially exports a subset of mRNAs encoding oncoproteins, such as Myc, CDC25A, BRD4, Bcl-2, Bcl-6, Mcl-1, Bcl-xL, cyclins, androgen receptor, and Pim1 by exporting several RNA-binding proteins, including LRPPRC, eIF4E, NXF3, and HuR; XPO1 thereby regulates the translation of these oncoproteins [[121], [122], [123], [124], [125], [126], [127], [128], [129]]; (3) exports U-snRNAs into the cytoplasm for modification and assembly, thus playing a critical role in the regulation of mRNA splicing through binding the adaptor protein PHAX; and (4) mediates the alternative export of both microRNAs and tRNAs.

### The role of XPO1 in cancer pathogenesis and drug-resistance

Altered nuclear export signaling is recognized as a driver of oncogenesis. XPO1 overexpression is observed in many types of cancers such as MM, pancreatic, gastric, prostate, and colorectal cancer [[130], [131], [132], [133], [134], [135], [136], [137], [138]]. In many of these cancers, XPO1 overexpression is induced by c-Myc and/or the loss of p53 [139]. XPO1 overexpression in cancer cells is associated with disease progression, treatment resistance, and inferior OS or PFS. Additionally, gain-of-function mutations of XPO1 (E571, R749, and D624) have been detected in many types of cancers, specifically in B-cell malignancies. For example, the XPO1^E571K^ mutation was detected in 33% of primary mediastinal B-cell lymphoma, 14% of classic Hodgkin lymphoma, 2% of DLBCL, and 3% of chronic lymphocytic leukemia [140]. XPO1^E571K^ mutation has been shown to cooperate with MYC and BCL2 in promoting lymphomagenesis. Furthermore, several XPO1 binding partners and adaptor proteins such as RAN, HuR, eIF4E, LRPPRC, and NXF3, are also frequently overexpressed in human cancers and correlate with a poor prognosis [120,[141], [142], [143], [144], [145], [146]]. Overexpression of XPO1 contributes to cancer pathogenesis through the nuclear-to-cytoplasmic export of tumor-suppressor proteins such as p53, BRCA1, P21, P27, APC, IκB, FOXO1A, FOXO3A, PP2A, maspin, and P63 [147,148]. These proteins are usually located in the nucleus and play their roles through regulating target gene expression. In addition, overexpression of XPO1 also results in cytosolic retention of survival proteins, including cIAP, surviving, and MCL1, as well as abnormal activation of survival and proliferation signaling,such as that of NF-κB and β-catenin, which are associated with disease progression.

**Fig. 3:**
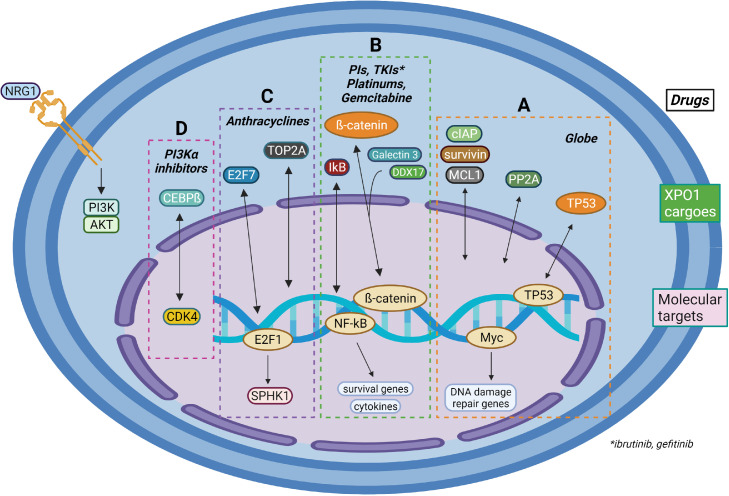
Molecular mechanisms by which XPO1 inhibitors restore drug sensitivity to resistant MM cells. Cytosolic retention of: A. TP53, cIAP/surviving/MCL1 and PP2A contribute to globe drug-resistance; B. galectin 3, DDX7 and IκB contribute to resistance of PIs, TKIs, platinum agents and gemcitabine due to the activation of β-catenin and NF-κB signaling. C. E2F7 and TOP2A cause resistance of anthracyclines. D. CEBPβ leads to resistance of PI3Ka inhibitors. Figure created with BioRender.com.

XPO1 has been identified as a critical player for drug resistance in many types of cancers [149,150]. Several molecular cargoes of XPO1 might contribute to global drug resistance, including tumor repressors (TP53, BRCA1, P21, and P27) and survival proteins (cIAP, survivin and MCL1). The elevated cytosolic levels of these proteins might be responsible for the resistance of many types of drugs [[151], [152], [153], [154], [155], [156], [157],^158^]. *In vitro* studies suggest that XPO1 promotes the expression of many recognized DNA damage repair proteins, including CHEK1, MLH1, MSH2, RAD51, and PMS2, most likely through a MYC-dependent fashion. Selinexor restores sensitivity to drug-resistant cells by retaining these substrates and repressing the expression of DNA-damage repair proteins (Fig. 3A).

Some of the cargoes of XPO1 may mediate drug-specific resistance. Correspondingly, this resistance may be overcome (Fig. 3B–D) by inhibiting XPO1. For example:

(1) In many cancer types, nuclear TOP2A inhibitor anthracycline resistance is driven by the cytosolic mislocalization of the transcriptional inhibitor E2F7 and topoisomerase TOP2. Mislocalization of E2F7 leads to the derepression of SPHK1; SPHK1 converts sphingosine to sphingosine-1-phosphate, conferring resistance to anthracyclines in these cancer types. Selinexor restores the anthracycline sensitivity in cancer cells by retaining E2F7 and TOP2A in the nucleus [[159], [160], [161], [162]].
(2) The increased cytosolic localization of β-galactosidase-binding protein galectin 3 has been linked with the resistance of platinum-based drugs such as cisplatin. This protein regulates the β-catenin signaling pathway through GSK-3β phosphorylation and is modulated by XPO1.
(3) Increased cytoplasm distribution of DDX17, an ATP-dependent DEAD box RNA helicase, has been linked to TKI gefitinib resistance in patients with EGFR-mutant non-small-cell lung cancers. DDX17 plays such a role by disassociating E-cadherin/β-catenin complexes, causing nuclear translocation of β-catenin.
(4) In PIK3CA-mutant ER^+^HER2^−^ metastatic breast cancers, an increased nuclear export of CEBP-β results in PI3K inhibitor-resistance due to CDK4-mediated cell cycle progression. In all these situations, inhibition of XPO1 can restore drug sensitivity by retaining corresponding cargoes in the nucleus.

In many types of MM cells, XPO1 has been defined as a critical player for resistance to both bortezomib and TOP2 inhibitors [[163], [164], [165], [166]]. Many XPO1-associated proteins including SMC1A, RCC2, CSE1, NUP88, NUP50, TPR, HSPA14, DYNLL1, RAD21, and RanBP2 are upregulated in bortezomib-resistant MM cells. XPO1 overexpression seems to be linked with bortezomib resistance and an unfavorable prognosis in MM patients. The subcellular localization of tumor suppressors or other key proteins may function as biomarkers of prognosis and/or treatment outcome. Selinexor restores the sensitivity of bortezomib and anthracyclines in cell line models, mouse models, and in patient-derived cells [165,167]. Selinexor plays this role via the nuclear retention of IκB, TOP2A, FOXO3a, and pro-survival proteins (cIAPs, Survivin and MCL1), as well as a down-regulation of MYC and its target genes [[165], [166], [167], [168], [169]]. Interestingly, the anti-tumor effects of selinexor are independent of the function of key tumor suppressor proteins RB, TP53, and p21 [[170], [171], [172]]. FOXO3a mediates selinexor-induced PUMA expression in a p53-independent fashion, explaining the clinical response of TP53-deficient MM to selinexor treatment [173].

### Selinexor in MM treatment

Selinexor is a small-molecule, first-in-class, oral SINE, which acts through blockading XPO1. Selinexor was granted accelerated approval by the FDA in July 2019 for penta-refractory MM and is now a category2A recommendation from the NCCN. It is also FDA approved along with bortezomib and dexamethasone as a third-line therapy. Although the efficacy of selinexor monotherapy (NCT01607892) was modest with only a 4% ORR and 21% clinical benefit rate [174], the effectiveness of selinexor and dexamethasone (Sd) combination greatly increased the ORR to 21-50%. STORM II was a phase II study which included 123 patients with RR-MM (median 7 prior lines of therapy); treatment with the Sd regimen (Selinexor 80 mg plus dexamethasone 20 mg twice weekly as part of a 28-day cycle) induced a total ORR in 26% of patients with a median duration of response of 4.4 months [39,40]. The median PFS and median OS were 3.7 and 8.8 months, respectively [39]. Based on this result, the Sd regimen was granted accelerated FDA approval in July 2019 for patients with MM who are refractory to at least 4 prior lines of therapy and whose diseases are refractory to at least 2 PIs, at least 2 IMiDS, and an anti-CD38 monoclonal antibody.

In a randomized phase III open-label BOSTON trial (KCP-330-023, NCT03110562) which included 402 patients with RR-MM (1–3 prior lines of therapy Vd [1.3  mg/m2 bortezomib and 20  mg dexamethasone twice weekly]) was compared to SelVd (selinexor 100  mg once weekly plus 1.3  mg/m2 bortezomib and 20  mg dexamethasone twice weekly). SelVd induced both an improved ORR of 76.4% vs 62.3% (*P*  = 0.0012) and improved PFS to 13.9 vs 9.5 months (*P*  = 0.0066) [40,175,176]. Based on this study, on December 18, 2020, the FDA approved selinexor in combination with bortezomib and dexamethasone for the treatment of adult patients with RR-MM who received at least one prior line of therapy. Detailed analysis of patient information and clinical parameters demonstrated that benefits of SelVd treatment over Vd were more pronounced in patients treated earlier in their disease course who had either received only one prior therapy, had never been treated with a PI, or had prior ASCT [177]. In addition, SelVd conferred benefits to patients over Vd regardless of cytogenetic risk [178]. Furthermore, SelVd is safe and effective in patients regardless of age and frailty scores [179].

### Adverse effects of selinexor

The most common grade 3 or 4 adverse events are hematologic, including thrombocytopenia, anemia, and neutropenia. The most common non-hematological adverse events are gastrointestinal disturbances including nausea, vomiting, decreased appetite, and diarrhea, as well as peripheral neuropathy, fatigue, upper respiratory tract infection, weight loss, which are all primarily grade 1 or 2 but can also be grade 3. Electrolyte imbalances, including asymptomatic hypophosphatemia, hyponatremia, and hypokalemia, have also been commonly observed [[175], [176], [177],^179^,^180^]. Approximately 19.5% of patients experienced ocular adverse events, including blurred vision and/or dry eye syndrome. Some patients showed progression of age-related nuclear sclerosis (cataract) [181]. Fortunately, all of these adverse events are manageable and generally reversible. Hematopoietic side effects are dose dependent and can be overcome by combining hematopoietic protective drugs and dose modifications [182]. However, most intestinal and non-hematological side effects seem associated with a brain neuronal response; therefore, development of SINEs with less blood-brain barrier penetration, such as KPT-8602, might be a potential solution [183].

## Perspectives moving forward

Several SINE compounds including KPT-185, KPT-249, KPT-251, KPT-276, KPT-330 (selinexor), KPT-335 (verdinexor), KPT-8602 (eltanexor), and SL-801 (felezonexor) are being investigated; however, selinexor is currently the only FDA approved SINE fo MM treatment. KPT-330, KPT-8602, and SL-80 have been advanced to early clinical trials. Clinical studies demonstrated that selinexor treatment only benefits a proportion of MM patients and has significant side effects. Some of the XPO1 cargoes such as HER2, YAP, and GSK3β might promote disease progress and drug resistance when accumulated in the nucleus [[184], [185], [186]]. Thus, to better use SINEs for MM treatment, it is important to (1) identify the predictive biomarkers (such as genetic abnormalities and/or gene expression profiling) to determine which patients are likely to respond to such inhibitors, avoiding unnecessary toxicity; (2) define novel and more effective selinexor combination regimens in order to improve response and reduce adverse effects; and (3) develop more selective and potentially less-toxic XPO1 inhibitors. KPT-8602 is a second-generation SINE [183] that retains XPO1 inhibitory activity as demonstrated in cell culture studies [187]. However, its penetration across the blood-brain barrier is 30-fold less than selinexor. It is suggested that KPT-8602 has less intestinal side effects and can offer a better tolerability profile while maintaining comparable efficacy.

Selinexor has been assessed in the preclinical setting for RR-MM and with other cancer treatments in combination with novel agents, including the Bcl2 inhibitor venetoclax, Bcl-xL inhibitors, azacitidine, tyrosine kinase inhibitors, topoisomerase inhibitors, the MDM2 inhibitor nutlin 3a, and immune checkpoint PD-1 inhibitors. However, these agents might preferentially benefit a certain subset of patients. For example, selinexor + venetoclax might benefit patients with t(11;14) who demonstrate high expression of BCL-2 [188]; selinexor + nutlin 3a might only benefit patients with at least one allele of TP53 expression; selinexor + azacitidine might benefit patients with mutations in epigenomic regulator genes such as EZH2 and PHF19; while selinexor + a MAPK inhibitor or a NF-kB inhibitor might benefit patients with mutations in KRAS/NRAS-MAPK and NF-κB pathways, respectively [189]. Thus, future studies need to focus on the correlation of genetic and epigenetic abnormalities to the specific combinations in order to develop more specific targeted therapies. While targeting therapeutic combinations based on individual patient genomic mutations appears attractive and has been shown to be effective in certain myeloid malignancies, branching evolutionary patterns at relapse, polyclonal disease, and increased mutational burden in general paralleling disease relapse will pose a challenge and necessitate multi-agent combinations moving forward [190].

Many MM patients acquire a “double-hit” of their TP53 gene (resulting in complete loss of TP53) after multple rounds of treatment and become resistant to most of the convertional myeloma therapeutics. Selinexor seems at least partially able to overcome this challenge by inducing TP53-independent apoptosis. However, “double-hit” mutations of the TP53 gene will still be an issue if a MM clone is unable to be completely eliminated. Different from TP53-expressing cancer cells, TP53-deficient cancer cells are entirely dependent on the G2/M checkpoints to maintain genome integrity and survival due to the tight feedback regulation of TP53 and mitotic kinases (eg, WEE1, PLK1, NEK2, BUB1, TTK, AURKB, and PLK1) [191,192]. Additonally, P53-deficient cells have also been reported to be dependent on the p38MAPK/MK2 pathway for survival following treatment with DNA-damaging agents. In consequence, TP53-deficient cancer cells are more sensitive to genotoxic stress when treated with inhibitors of these kinases [[193], [194], [195], [196], [197]]. Future studies need to test whether selinexor can more effectively kill P53-deficient MM cells when combined with the selective inhibitors these kinases.

## Funding

This work was supported by 10.13039/100000135NIH grants R01 HL133560-01 and R01 CA223194-01 through Loyola University Chicago, Loyola program development funds to Jiwang Zhang, and translational development grants through the Cardinal Bernardin Cancer Center to Stephanie Berg, Patrick Hagen, and Jiwang Zhang.

## Ethics approval

This is not applicable for this review.

## Consent to participate

This is not applicable for this review.

## Consent for publication

This is not applicable for this review.

## Data availability statement

Data sharing is not applicable to this article as no new data were created or analyzed in this study.

## Code availability

This is not applicable for this review.

## Clinical trial registration

This is not applicable for this review.

## CRediT authorship contribution statement

**Mark Sellin:** Writing – original draft, Writing – review & editing, Visualization, Conceptualization. **Stephanie Berg:** Writing – original draft, Writing – review & editing, Visualization, Conceptualization. **Patrick Hagen:** Writing – original draft, Writing – review & editing, Visualization, Conceptualization. **Jiwang Zhang:** Writing – original draft, Writing – review & editing, Visualization, Conceptualization.

## Declaration of Competing Interest

None.
